# The prevalence and correlates of physical inactivity among adults in Ho Chi Minh City

**DOI:** 10.1186/1471-2458-8-204

**Published:** 2008-06-09

**Authors:** Oanh TH Trinh, Nguyen D Nguyen, Michael J Dibley, Philayrath Phongsavan, Adrian E Bauman

**Affiliations:** 1 Faculty of Public Health, University of Medicine and Pharmacy, Ho Chi Minh City, Vietnam; 2 School of Public Health and the George Institute for International Health, University of Sydney, NSW 2006, Australia; 3 Centre for Physical Activity and Health, School of Public Health, University of Sydney, NSW 2006, Australia

## Abstract

**Background:**

Socioeconomic changes have led to profound changes in individuals' lifestyles, including the adoption of unhealthy food consumption patterns, prevalent tobacco use, alcohol abuse and physical inactivity, especially in large cities like Ho Chi Minh City (HCMC). The Stepwise Approach to Surveillance of Non-communicable Disease Risk Factors survey was conducted to identify physical activity patterns and factors associated with 'insufficient' levels of physical activity for health in adults in HCMC.

**Methods:**

A cross-sectional survey was conducted in 2005 among 1906 adults aged 25–64 years using a probability proportional to size cluster sampling method to estimate the prevalence of non-communicable disease risk factors including physical inactivity. Data on socioeconomic status, health behaviours, and time spent in physical activity during work, commuting and leisure time were collected. Physical activity was measured using the validated Global Physical Activity Questionnaire (GPAQ). Responders were classified as 'sufficiently active' or 'insufficiently active' using the GPAQ protocol. Correlates of insufficient physical activity were identified using multivariable logistic regression.

**Results:**

A high proportion of adults were physically inactive, with only 56.2% (95% CI = 52.1–60.4) aged 25–64 years in HCMC achieving the minimum recommendation of 'doing 30 minutes moderate-intensity physical activity for at least 5 days per week'. The main contributors to total physical activity among adults were from working and active commuting. Leisure-time physical activity represented a very small proportion (9.4%) of individuals' total activity level. Some differences in the pattern of physical activity between men and women were noted, with insufficient activity levels decreasing with age among women, but not among men. Physical inactivity was positively associated with high income (OR = 1.77, 95% CI = 1.05–2.97) and high household wealth index (OR = 1.86, 95% CI = 1.29–2.66) amongst men.

**Conclusion:**

Public health policies and programs to preserve active commuting in HCMC and to promote time spent in recreational physical activity in both genders and across all age groups, but especially among young adults, will be critical in any comprehensive national plan to tackle inactivity. Clear and consistent national recommendations about how much physical activity Vietnamese people need for preventing and managing non-communicable diseases should also be part of this population-wide promotional effort.

## Background

During recent decades, epidemiological studies have indicated that physical inactivity is associated with a variety of non-communicable diseases (NCDs) and risk factors, such as obesity, heart disease, and cancer [1]. According to the World Health Organization (WHO), physical inactivity is estimated to cause, globally, about 10–16% of cases of breast, colon and rectal cancers and diabetes mellitus, and about 22% of ischaemic heart disease. Overall, 1.9 million deaths are attributable to physical inactivity [2]. Countries in the South-East Asia region are going through an epidemiological transition, and NCDs account for up to 51% of all deaths and 44% of the disease burden in this region [3]. The shift towards industrialization and urbanization in lower-income countries from agricultural labor towards employment in manufacturing and services implies a reduction in energy expenditure [4].

Following the social and economic policy reforms of 1986, Vietnam is considered as an emerging economy in South East Asia with the Gross Domestic Product increasing by over 7% per year [5]. The resulting changes in the economy and consequently in society have led to profound changes in individuals' lifestyles, including the adoption of unhealthy food consumption patterns, prevalent tobacco use, alcohol abuse and physical inactivity, especially in large cities like Ho Chi Minh City (HCMC). As a consequence, the epidemiological pattern of diseases has changed dramatically in the past 20 years with morbidity from increasing NCDs [6] forecast as important public health problems in the coming years [7]. Cuong reported that HCMC populations were suffering a double burden of not only underweight but also overweight and obesity [8]. The prevalence of overweight and obesity in HCMC using the WHO body mass index (BMI) cut-off values recommended for Asian countries [9] (BMI ≥ 23 kg/m^2 ^and ≥ 27.5 kg/m^2 ^for overweight and obesity, respectively) were 26.2% and 6.4% respectively [8].

In 2002 Vietnam launched the first national program for NCD prevention and control (Vietnamese National Health Strategy 2001–2005). It was agreed that epidemiological studies of health risk behaviours would provide important information for health policy makers in HCMC. A 'Stepwise Approach to Surveillance of Non-communicable Disease Risk Factors Survey' (commonly known as STEPwise survey)[10] was carried out in 2005 to provide a first snapshot of NCD-related risk factors among adults aged 25–64 years living in HCMC. The standardised STEPwise questionnaire was used and findings from the physical activity component of this survey are presented here. We report on the prevalence of physical activity among adults, the time they spent engaging in moderate- and vigorous-intensity activities during work, commuting and recreation, and the identification of groups at risk of physical inactivity. To our knowledge, no study of physical activity has been conducted with a population-based sample of adults in Vietnam that focuses on these three important domains of individuals' activity level. Findings from this study will provide a baseline against which the national program for the prevention and control of NCDs can be monitored.

## Methods

### Study population

This was a cross-sectional study of a representative sample of Vietnamese adults aged 25–64 years living in HCMC. The sample size was calculated to yield prevalence estimates for NCD risk factors with the expected precision of ± 8%. A total of 1981 of the 2355 invited adults aged 25–64 years participated in the study (response rate 84.1%). After eliminating records that had missing information on physical activity (for each domain or all, 70 records) or over-reported on total of minutes spent in physical activity per day (> 1440 minutes/day, 5 records), the final usable sample size was 1906 (missing 3.8%). There were no significant differences in socio-demographic characteristics between the usable sample and the respondents with missing physical activity data (p > 0.05).

### Survey sampling strategy

The probability proportional to size cluster sampling (PPS method) was used to select the study sample[10]. The sampling frame comprised a list of 317 wards/communes in HCMC. Wards/communes were the primary sampling units and sixteen wards/communes were selected using the PPS method. In each ward/commune, a list of all adults aged 25–64 years was identified from the 2004 CENSUS for HCMC, which was provided by the local government. Prior to selecting participants for each ward/commune, data from the lists were entered into the computer and stratified by sex and age groups. There were eight age-sex groups: 25–34 years, 35–44 years, 45–54 years and 55–64 years, with 16 persons selected from each age-sex group using systematic random sampling. Therefore, 128 adults in each ward/commune were selected. As well as the main lists, reserved lists were also generated at the same and in the same manner. That is, the probability of a person being selected in both lists was the same. Selected participants from the main list who did not consent or were ineligible (due to physical or mental disabilities, deceased or moved out of ward/commune) were replaced by persons from the same sex-age group in the reserve lists. The reserve lists were necessary to ensure that the study achieved the required sample size for each stratum. The proportion of replacements was 15.5% of consented individuals.

The final lists of potential participants were sent to local health workers who were responsible for approaching and inviting participants. All participants received an information sheet about the study and a letter inviting their participation in the study. If they agreed, participants would then be asked to sign a consent form and arrangements were made to schedule their visit to the local health centre for the survey. Participants were interviewed in person by well-trained interviewers from the Faculty of Public Health.

The study protocol as well as ethical issues were cleared and approved by the Faculty of Public Health and the University of Medicine and Pharmacy of Ho Chi Minh in Vietnam. Before the study commenced in the field, the procedure was also approved by the local government as well as the local health centre. Local government authorities and health workers played an important role in providing the lists of potential participants, and inviting and motivating participants to be involved the study.

### Physical activity measure

The physical activity measure used was the Global Physical Activity Questionnaire (GPAQ) [11] which comprised 19 questions about physical activity performed in a typical or usual week. The GPAQ measure asked about the frequency (days) and time (minutes/hours) spent doing moderate- and vigorous-intensity physical activity in three domains: [i] work-related physical activity (paid and unpaid including household chores), [ii] active commuting (walking and cycling), and [iii] discretionary leisure-time (recreation) physical activity. GPAQ is an instrument derived from the long and short forms of the IPAQ (International Physical Activity Questionnaire) which has been validated and widely used to assess physical activity patterns [12]. The test re-test reliability of GPAQ (short-term assessment in 3- to 7-day interval) produced good-to-excellent results (r = 0.67–0.81) and the concurrent validity against IPAQ for total physical activity yielded a moderate-to-good correlation (r = 0.54) and for sedentary questions generated a good correlation (r = 0.65)[13].

No changes were made to the original contents and wording of the questionnaire following the translation of the measure from English to Vietnamese. However, local examples of types and intensity of activities were used to suit the Vietnamese context. All data collection and processing followed the GPAQ analysis protocol [11].

#### Physical activity data treatment, definitions and analysis

Energy expenditure was estimated based on the duration, intensity and frequency of physical activities performed in a typical week. The unit for measuring physical activity energy expenditure, Metabolic Equivalent (MET), was applied to physical activity variables derived from the GPAQ. MET is the ratio of specific physical activity metabolic rates to the resting metabolic rate. One MET is equivalent to the energy cost of sitting quietly (1 kcal/kg/hour) and oxygen uptake in ml/kg/min with one MET is equal to the oxygen cost of sitting quietly, around 3.5 ml/kg/min. MET values and formulas for computation of MET-minutes are based on the intensity of specific physical activities: a moderate-intensity activity during work, commuting and recreation is assigned a value of 4 METs; vigorous-intensity activities are assigned a value of 8 METs. The total physical activity score is computed as the sum of all MET/minutes/week from moderate- to vigorous-intensity physical activities performed in work, commuting and recreation [11].

Physical activity levels were initially classified into low, moderate or high (vigorous) intensity as defined by the GPAQ analysis framework [11]:

(1) **High: **Any one of the following two criteria: (a) vigorous-intensity activity on at least 3 days and accumulating at least 1500 MET-minutes/week OR (b) 7 or more days of any combination of walking, moderate- or vigorous-intensity activities accumulating at least 3000 MET-minutes/week.

(2) **Moderate**: Either of the following three criteria: (a) 3 or more days of vigorous-intensity of at least 20 minutes per day OR (b) 5 or more days of moderate-intensity and/or walking of at least 30 minutes per day OR (c) 5 or more days of any combination of walking, moderate-or vigorous-intensity activities accumulating at least 600 MET-minutes/week.

(3) **Low: **No activity is reported or some activity is reported but not enough to meet high and moderate categories.

These three groupings were then categorized into 'sufficiently active' or 'insufficiently active' groups. The 'sufficiently active' group included participants who met the physical activity recommendation, therefore classified as being in the moderate or high (vigorous) intensity category.

No physical activity during work, commuting and recreation were determined based on the yes/no questions: '*Does your work involve mostly sitting or standing, with walking for no more than 10 minutes at a time*?' (working time), '*Do you walk or use a bicycle for at least 10 minutes continuously to get to and from places?' *(commuting time), and '*Does your recreation, sports or leisure time involve mostly sitting, reclining, or standing, with no physical activities lasting more than 10 minute at a time?' *(leisure time).

### Socio-demographic variables

Socio-demographic variables measured age, gender, education level, occupation, location of residence, monthly household income, and number of household appliances.

Household wealth index was defined based on household appliances as a measure of economic status. Household appliances listed were: vehicles (bicycle/boat, motorcycle/motorbike, car/truck), entertainment appliances (radio/cassette players, television, CD/VCD/DVD, cable TV, computer, video-game) and other household appliances (rice cooker, fan, gas oven, magnetic oven, washing machine, refrigerator, and air-conditioner). This list was constructed using the methods recommended by the World Bank Poverty Network and UNICEF, and described by Filmer & Pritchett [14]. The wealthy index was then computed by grouping households into quintiles, from the poorest to the richest.

Data on smoking status and alcohol consumption were also collected. Smoking status was classified as current smoker, ex-smoker, and non-smoker. Binge alcohol consumption was defined as having 5 or more standard drinks per day and 4 or more standard drinks per day for men and women, respectively.

### Statistical analysis

Data were weighted using post-stratified weights to adjust for stratification data during sampling. Although PPS sampling method was self-weighted, post-stratified weights were calculated based on the population distribution of adults aged 25–64 years for both genders living in HCMC (reference population from 2004 CENSUS for HCMC). Epidata was used to enter data and all analyses were performed using Stata/SE software version 9.2, with the *svyset *commands used to compute standard errors for surveys with stratified cluster sample.

#### Descriptive statistics

The prevalence of levels of physical activity and other categorical variables are reported as proportions with 95% confidence interval (CI). Continuous variables such as time spent in physical activity are reported as median (50^th^) and inter-quartile range (25^th^, 75^th^) due to their skewed distributions. Mean values are also reported for additional information.

#### Analytic statistics

Chi-squared test (Pearson chi-squared) was performed to test the relationship between socio-demographic and physical activity variables at a significance level of 0.05. Tests for linear trend across categories are reported when examining dose-response relationships. Univariate logistic and multivariable logistic models were used to estimate odds ratios (ORs) and to control for potential confounders as well as modelling interaction terms. Collinearity among education, income and wealth index was examined and found to be < 0.5. Because crude and adjusted ORs were almost similar, only adjusted ORs are reported. The Wald test is reported at a significance level of 0.05.

## Results

### Population characteristics

Table 1 shows no differences in the weighted sample distribution by gender and across age, area, and ethnicity. The age group distribution was similar to the population distribution of HCMC (i.e. 2004 CENSUS). In general, the proportion of participants in each socio-demographic category was large enough to perform tests and models except for the ethnicity variable (category 'other' comprising 4.3% of the sample).

**Table 1:** Characteristics of the survey sample, by gender*

	**Male (n = 884) n (%)**	**Female (n = 1022) n (%)**	**Both (n = 1906) n (%)**
**Gender**	884 (47.7)	1022 (52.3)	1906 (100.0)
**Age groups**			
25–34	367 (41.5)	400 (39.1)	766 (40.2)
35–44	286 (32.3)	325 (31.8)	612 (32.1)
45–54	169 (19.1)	205 (20.1)	374 (19.6)
55–64	62 (7)	92 (9)	154 (8.1)
Area**			
Wealthy urban	201 (22.7)	249 (24.4)	450 (23.6)
Less wealthy urban	455 (51.5)	514 (50.3)	970 (50.9)
Suburban	228 (25.8)	259 (25.3)	486 (25.5)
**Ethnic**			
Kinh	850 (96.2)	976 (95.5)	1826 (95.8)
Others	34 (3.8)	46 (4.5)	80 (4.2)
**Education**^a^			
	*(n = 883)*	*(n = 1021)*	*(n = 1904)*
Less than primary school	90 (10.2)	168 (16.4)	258 (13.4)
Primary school completed	258 (29.2)	333 (32.6)	591 (31.0)
Secondary school completed	227 (25.7)	254 (24.9)	481 (25.3)
High school completed	178 (20.2)	162 (15.9)	340 (17.9)
Some colleges	130 (14.8)	104 (10.2)	234 (12.4)
**Occupation**^a^			
	*(n = 881)*	*(n = 1021)*	*(n = 1902)*
Government employee	124 (14.1)	129 (12.7)	253 (13.3)
Non-Government employee	259 (29.4)	169 (16.6)	428 (22.7)
Self-employee	366 (41.6)	333 (32.6)	699 (36.9)
Housewife	3 (0.3)	344 (33.7)	337 (17.8)
Others (unpaid, retired, student, unemployed)	129 (14.6)	46 (4.5)	175 (9.3)
**Household economic status**			
*Income/month*†^a^	*(n = 832)*	*(n = 960)*	*(n = 1792)*
< 1,000,000	121 (14.5)	180 (18.8)	301 (16.7)
1,000,000 -< 3,000,000	403 (48.5)	502 (52.3)	905 (50.5)
3,000,000 -< 5,000,000	154 (18.5)	143 (14.9)	297 (16.6)
≥ 5,000,000	154 (18.5)	135 (14.0)	289 (16.2)
*Household wealth index*^b^			
	*(n = 881)*	*(n = 1021)*	*(n = 1902)*
Lowest	154 (17.5)	238 (23.3)	390 (20.5)
Second	179 (20.3)	209 (20.5)	388 (20.4)
Middle	195 (22.1)	181 (17.7)	377 (19.8)
Fourth	181 (20.6)	201 (19.7)	382 (20.1)
Highest	172 (19.6)	192 (18.8)	365 (19.2)
**Tobacco use**^c^			
Non-smoker	209 (23.6)	997 (97.6)	1187 (62.3)
Ex-smoker	166 (18.8)	8 (.8)	179 (9.4)
Current smoker	509 (57.5)	16 (1.6)	540 (28.3)
**Alcohol consumption**^c^			
	*(n = 880)*	*(n = 1020)*	*(n = 1900)*
Non-binge drinking	622 (70.7)	1009 (98.9)	1631 (85.4)
Binge drinking‡	258 (29.3)	11 (1.1)	269 (14.6)

* Data weighted for age and gender based on the national 2004 CENSUS

**Classification based on the HCMC Bureau of Statistics, 2002

† General income of household in millions VND

‡ 5 standard drinks or more for men and 4 standard drinks or more for women

^a ^Pearson chi-squared test with p < 0.001, ^b ^p < 0.01, ^c ^p < 0.05

### Time spent in physical activity

Based on quintile values (25^th^, 50^th^, and 75^th^) and the recommended physical activity level, at least 50% of participants were insufficiently active in each domain with the majority of physical activity time emanating mostly from working and active commuting, especially among women (Table 2). It is interesting to note that minutes spent in recreational physical activity was close to zero, with at least 75% of participants doing no physical activity in their leisure time. This pattern was similar by gender and age groups.

**Table 2:** Median and mean minutes spent per day at work, commuting and recreation in adults aged 25–64 years

	**Age groups**	**25–34**	**35–44**	**45–54**	**55–64**	**25–64**
**Men (n = 884)**						
Working	Mean	121.6	119.2	77.1	50.6	107.3
	Median	0	0	0	0	0
	(25^th^, 75^th^)	(0, 205.7)	(0, 205.7)	(0, 51.4)	(0, 0)	(0, 68.6)
Commuting	Mean	29.3	35.5	32.9	46.9	33.2
	Median	0	0	0	15	0
	(25^th^, 75^th^)	(0, 30)	(0, 30)	(0, 30)	(0, 60)	(0, 30)
Recreation	Mean	9.5	2.3	8.60	5.0	6.7
	Median	0	0	0	0	0
	(25^th^, 75^th^)	(0, 0)	(0, 0)	(0, 0)	(0, 0)	(0, 0)
**Women (n = 1022)**						
Working	Mean	73.4	95.7	99.0	73.8	85.6
	Median	0	0	0	0	0
	(25^th^, 75^th^)	(0, 10)	(0, 105)	(0, 120)	(0, 60)	(0, 10)
Commuting	Mean	30.7	34.2	45.4	46.9	36.2
	Median	7.5	17.1	21.4	30	20
	(25^th^, 75^th^)	(0, 30)	(0, 45)	(0, 60)	(0, 60)	(0, 51.4)
Recreation	Mean	2.3	4.2	2.6	4.5	3.2
	Median	0	0	0	0	0
	(25^th^, 75^th^)	(0, 0)	(0, 0)	(0, 0)	(0, 0)	(0, 0)
**Both (n = 1906)**						
Working	Mean	97.1	106.9	88.8	64.2	96.0
	Median	0	0	0	0	0
	(25^th^, 75^th^)	(0, 60)	(0, 120)	(0, 102.9)	(0, 30)	(0, 60)
Commuting	Mean	30.0	34.8	39.6	46.9	34.8
	Median	0	10	15	20.0	12.9
	(25^th^, 75^th^)	(0, 30)	(0, 34.3)	(0, 51.4)	(0, 60)	(0, 42.9)
Recreation	Mean	5.8	3.3	5.4	4.7	4.8
	Median	0	0	0	0	0
	(25^th^, 75^th^)	(0,0)	(0,0)	(0,0)	(0,0)	(0, 0)

Physical activity patterns were different by gender for work and for the active commuting domains. At the 75^th ^percentile, minutes worked were higher in younger men and decreased rapidly in middle-age. However, the upper quartile for young men shows high work-related activity (> 200 minutes/day) and this amount declined to 0 for at least 75% of participants aged 55 years and older. Whereas the upper quartile point for minutes of work-related activity among women increased steadily with increasing age and only reduced among those aged 55–64 years, but this was still higher than men in the same age group. Time spent in active commuting among women increased with age, but was relatively stable in the three younger age groups of men and increased only in the oldest group. The mean values in each domain also indicated the same pattern as median results.

### Being sufficiently active for health

**Figure 1:**
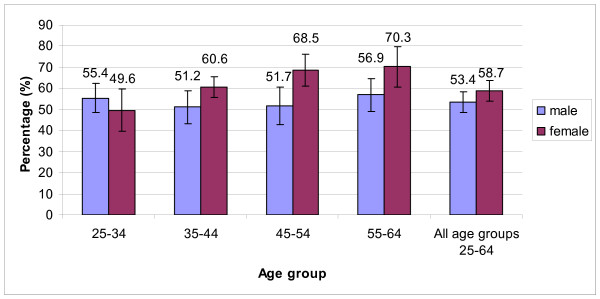
Prevalence of adults being sufficiently active for health by age and gender in HCMC, Vietnam.

Overall, 56.2% (95% CI = 52.1–60.4) of adults aged 25–64 years in HCMC were 'sufficiently active' and this prevalence increased with increasing age. Figure 1 revealed that women were generally more active than men (58.7% and 53.4%, respectively). Although the proportion of active women aged 25–34 years was lower than men, the proportion increased substantially from 49.6% in the youngest group to 70.3% in the oldest group (p < 0.01). Among men, there were some fluctuations between 51.2% and 56.9% across the age groups (p > 0.05) (Figure 1). Time spent engaging in physical activity during work and commuting increased continuously with age in women, and this contributed to a higher 'sufficiently active' prevalence among women. However, the pattern of physical activity in recreation time was similar for all ages and genders, and contributed very little to total physical activity in this population (p > 0.05).

### Patterns of no physical activity during work, commuting and leisure

**Figure 2:**
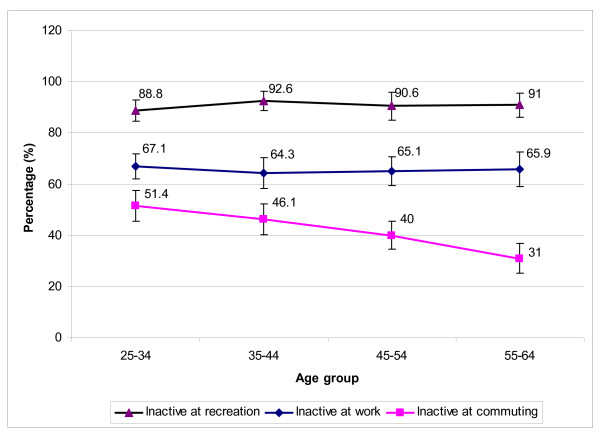
Distribution of participants classified as doing no work-commuting-recreational-related physical activity by age group.

In general, the proportions classified as doing no physical activity at work and during leisure time were not different across ages and varied between 64.3% to 67.1% and from 88.8% to 92.6% for work and leisure time, respectively (p > 0.05) (Figure 2). With regard to active commuting, the percentage of inactive people declined with increasing age. Reports of 'no active commuting' decreased from 51.4% in the youngest age group to 31% in the oldest group (p < 0.01). Figure 2 also shows that the youngest group (25–34 years old) was the most passive group with respect to the three domains (highest inactive rates).

**Figure 3:**
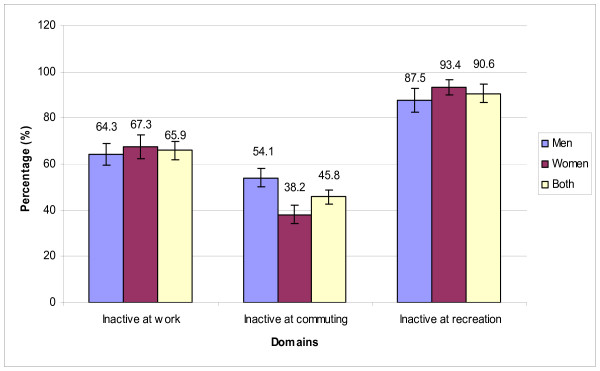
Distribution of participants classified as doing no work-commuting-recreational-related physical activity by gender and overall.

Figure 3 suggests that although the percentage of men classified as doing no physical activity-related work and no active recreation was lower than women, proportionately more women than men engaged in active commuting (reporting transport activity in 62% compared to 45.9%, respectively). However, the difference between genders was only significant for commuting (p < 0.0001). From Figure 2 and Figure 3, we can see that recreation was the most passive domain and commuting represented the most active domain, especially for women.

### Social-demographic correlates of insufficient physical activity

Results in Table 3 indicate that only income, household wealth index, and smoking were significantly related to insufficient physical activity. Monthly income of more than 1 million Vietnamese Dong (VND) was associated with insufficient activity. This association was significant for the groups with 1–3 million VND and more than 5 million VND. However, the household wealth index shows a significant association from the middle quintiles onwards, with people from wealthier households having greater risks of insufficient activity, especially among men. Tests for trend across income and household wealth index also confirmed this observation (p < 0.001). Although the results across both genders show this strong association, we did not see any significant association in women. Risks of insufficient activity in the non-smoker group was higher than ex-smokers and current smokers with OR = 0.58 (95% CI = 0.37–0.91) and OR = 0.76 (95% CI = 0.54–1.05), respectively.

**Table 3:** Association between socio-economic characteristics and insufficient physical activity by gender in adults aged 25–64 years in HCMC^a^

	**Male (n = 821) Adjusted OR (95%CI)**	**Female (n = 955) Adjusted OR (95%CI)**	Both (n = 1776)** Adjusted OR (95%CI)
**Gender**			
Men	-	-	ref.
Women	-	-	0.79 (0.54–1.17)
**Age groups**^c^			
25–34	ref.	ref.	ref.
35–44	1.31 (0.82–2.11)	0.77 (0.47–1.26)	0.98 (0.71–1.34)
45–54	1.21 (0.73–2.03)	0.64 (0.41–1.00)	0.86 (0.66–1.11)
55–64	0.98 (0.56–1.69)	0.64 (0.33–1.23)	0.74 (0.52–1.06)
Area*			
Wealthy urban	ref.	ref.	ref.
Less wealthy urban	1.24 (0.90–1.71)	1.20 (0.79–1.82)	1.23 (0.90–1.68)
Suburban	1.09 (0.60–1.98)	1.20 (0.81–1.77)	1.16 (0.76–1.77)
**Ethnic**			
Kinh	ref.	ref.	ref.
Others	1.27 (0.27–5.99)	1.80 (0.58–5.63)	1.62 (0.90–2.95)
**Education**^c^			
Less than primary school	ref.	ref.	ref.
Primary school completed	0.86 (0.38–1.94)	0.83 (0.45–1.52)	0.88 (0.56–1.37)
Secondary school completed	0.83 (0.36–1.92)	0.91 (0.45–1.85)	0.93 (0.55–1.56)
High school completed	0.95 (0.51–1.78)	1.08 (0.58–2.02)	1.09 (0.68–1.75)
Some colleges	1.1 (0.46–2.62)	1.46 (0.49–4.30)	1.32 (0.63–2.76)
**Occupation**^c^			
Government employee	ref.	ref.	ref.
Non-Government employee	0.96 (0.42–2.19)	1.86 (1.0–3.45)	1.24 (0.77–2.0)
Self-employee	1.40 (0.58–3.36)	1.11 (0.63–1.97)	1.27 (0.82–1.96)
Housewife	-	0.69 (0.41–1.17)	0.65 (0.36–1.17)
Others (unpaid, student, unemployed, retired)	1.05 (0.49–2.23)	0.69 (0.24–1.98)	0.97 (0.57–1.64)
**Household economic status**^c^			
*Income/month *†			
< 1,000,000	ref.	ref.	ref.
1,000,000 -< 3,000,000	1.57 (0.94–2.62)	1.44 (0.88–2.35)	**1.42 (1.02–2.00)**^b^
3,000,000 -< 5,000,000	**1.98 (1.04–3.74)**^b^	1.30 (0.56–3.0)	1.51 (0.94–2.43)
≥ 5,000,000	2.0 (.96–4.16)	1.71 (0.85–3.45)	**1.77 (1.05–2.97)**^b^
*Household wealth index*^c^			
Lowest	ref.	ref.	ref.
Second	1.32 (0.65–2.67)	1.23 (0.75–2.01)	1.29 (0.9–1.84)
Middle	**1.82 (1.03–3.2)**^b^	1.44 (0.76–2.72)	**1.67 (1.26–2.21)**^b^
Fourth	1.68 (0.78–3.66)	1.92 (0.96–3.86)	**1.87 (1.15–3.04)**^b^
Highest	**2.0 (1.23–3.25)**^b^	1.66 (0.72–3.82)	**1.86 (1.29–2.66)**^b^
**Tobacco use**^d^			
Non-smoker	ref.	ref.	ref.
Ex-smoker	**0.57 (0.36–0.90)**^b^	0.42 (0.03–6.86)	**0.58 (0.37–0.91)**^b^
Current smoker	0.75 (0.52–1.08)	0.39 (0.05–3.06)	0.76 (0.54–1.05)
**Alcohol consumption**^d^			
Non-binge drinking	ref.	ref.	ref.
Binge drinking‡	1.05 (0.69–1.62)	0.73 (0.21–2.5)	1.02 (0.71–1.47)

^a ^OR adjusted for all variables in the table 3; ^b ^p < 0.05 (Wald test); ^c ^p < 0.001 (test for trend); ^d ^p < 0.05 (test for trend)

* Classification based on the HCMC Bureau of Statistics, 2002; ** Missing data due to refusal;

† General income of household in millions VND; ‡ 5 standard drinks or more for men and 4 standard drinks or more for women

Other variables such as age, education level, occupation, ethnicity and area also showed an association with insufficient activity, but were not significant (Table 3). However, tests for trend across age, education and occupation indicated that the older and the more educated an individual, the more inactive they were (p < 0.001). While the OR increased with age in men, age was a protective factor for women. Associations between location, alcohol consumption, ethnic group and insufficient physical activity were not evident.

## Discussion

Over the last two decades there has been considerable interest in the impact of rapid social and economic developments on health-related behaviours. The present study is the first effort to systematically gather epidemiological evidence that focuses exclusively on population-level physical activity patterns and the correlates of insufficient physical activity among Vietnamese adults living in HCMC. Accurately assessing the prevalence of physical inactivity is an important component of non-communicable disease prevention, especially in countries with rapid lifestyle transitions as a consequence of economic progress.

This study shows that 56% of adults in HCMC are physically active, that is meeting the minimum recommendation of 30 minutes of moderate-intensity physical activity for 5 or more days per week. The prevalence is similar to Brazil [15], but lower than that in urban areas in China [16]. Consistent with findings from other studies in developing countries [15,17,18], our results also show that occupational activity and active commuting are the main contributors to total physical activity among adults in HCMC, implying that the surveyed population still engaged in labour intensive occupations and used active forms of commuting to and from places (cycling, walking). These findings highlight two key issues for consideration. First, assuming that continuing growth in the Vietnamese economy will result in significant urbanisation of the environments and infrastructure and a shift to occupations that are more sedentary, it is postulated that the prevalence of overall physical activity may decline as the country becomes more developed. Given that the behavioural patterns of the population could be significantly altered, a systematic promotion of physical activity and its health-enhancing benefits should be regarded as a high public health priority.

Second, although several epidemiological studies have demonstrated the importance of work and active commuting as key sources of energy expenditure and have highlighted their potential contributions to health [19-23], these forms of physical activity are not routinely measured compared to other forms of activity in routine physical activity surveys. Assessments of active commuting [22,24] and activities relating to work and domestic activities [19,20,23] should be an important part of physical activity surveillance in Vietnam.

In addition, physical activity undertaken as part of recreational or leisure-time activity contributed very little (9.4%) to the overall physical activity level in this population. A similar pattern is seen in other countries in the region. For example, 14% of Taiwanese adults aged 20 years or older [18] and 7.9% of adults in China [16] engaged in leisure-time physical activity. In developed countries, leisure-time physical activity is a major component of total physical activity undertaken by adults [25,26]. When comparing leisure-time physical activity of the youngest age group in the survey (25–34 years) with an international data of university students aged 17–30 years in developed and developing countries [27], the proportion of inactivity in the former group was double (88.8% compared to 44% in the developing country group and 42% in Pacific/Asian group). This difference may reflect a higher availability and accessibility to sports or recreational facilities as well as organised physical activity programs or sports curricular in universities. Since leisure-time physical activity is not common in Vietnam, it is unlikely that such activities will replace occupation or commuting activities in the immediate future. Therefore, developing countries that focus only on promoting leisure-time physical activity might not reduce the level of physical inactivity and under-value health-enhancing physical activities that might be undertaken as part of active commuting and working among adults.

The high prevalence of insufficient physical activity observed across all age groups and genders, especially during recreation, could reflect limited access to and availability of leisure-time physical activity. The findings (Table 2) observed in this study further suggest that the surveyed populations were already meeting the current physical activity recommendations through work and commuting. This could explain the contradictory findings of why more than 50% of people were found to be inactive in each domain (median minutes = 0), especially in leisure-time activity, whilst the overall percentage of 'sufficient physical activity for health' in this population was 56.2%. However, this pattern could also reflect a polarization in physical activity and inactivity behaviours of the HCMC populations which comprise of populations that are inactive during work, commuting and leisure time and other populations that are generally active but mainly through work and active commuting. This highlights the importance of documenting the population-level prevalence of physical activity and inactivity in each of the physical activity domains. A better understanding of these domains and their correlates has the potential to inform public health programs aimed at promoting physical activity and decreasing time spent on sedentary activities.

Some important differences in physical activity patterns between Vietnamese men and women were observed. Through active commuting (and to some extent occupational activities) women were more active than men and continued to be more active with increasing age. These two domains contributed considerably to the overall physical activity levels in women, especially for those in the three older age groups compared to similarly aged men. These results are contrary to findings from other countries where physical activity levels among women were reported to be lower than those in men [16,24,28], with prevalence rates often reduced with increasing age [16,17,24,28,29]. This could be explained by the high proportion of women doing domestic activities (33.7% of women compared to 0.3% of men), who are of lower education and lower income, and who therefore would be unlikely to own a motorbike. The routine of walking to the market daily (about 0.5 km from home), or taking a motorbike to the market, but then, after parking the motorbike, women might walk around the market. This could have also contributed substantially to women maintaining an active lifestyle.

Evidence from several national surveys in developing countries suggests that the prevalence of insufficient physical activity increased with increasing socio-economic status levels [15-17,27,28]. This is in contrast to physical activity patterns seen in developed countries [26,30,31]. In this study, high income, high household wealth index, and smoking were significantly associated with insufficient activity, especially for men. No strong associations were found between insufficient activity and various socio-demographic variables. However, tests for linear trend indicated significant associations between insufficient activity with higher levels of education, sedentary occupations, younger age, less wealthy areas and ethnicity (Chinese, Khmer). This is in contrast to other studies showing that while active commuting and work-related physical activity are more prevalent among the poor, leisure-time physical activity is more common among the rich [15]. This suggests that for some populations in HCMC being wealthy, being more educated and having low activity occupations, and being of younger age also implied a higher risk of adopting a physically inactive lifestyle. These unique patterns of relationships between various socio-demographic factors and insufficient physical activity will necessitate carefully tailored public health programs targeting more affluent and educated population groups.

Although current smoking was not significantly associated with physical activity, the results did indicate a lower risk for insufficient activity (borderline significance). This result contradicts findings by other studies [24,25]. A possible explanation for this observation is confounding by occupational physical activity, where smoking is highest amongst men engaging in labour-intensive occupations compared to women (57.5% in men and 1.6% in women). Furthermore, a person may give up smoking due to adverse health status, and this might then lead to increased physical activity.

We acknowledge that certain factors might influence the findings of the current study. Firstly, over-reporting or problems with recall cannot be dismissed in self-reporting measures. For example, over-reporting of physical activity may occur due to recall or social desirability, which would lead to overestimating the prevalence of sufficient physical activity. Second, test-retest and validity of the IPAQ measure suggested that its reliability and validity were lower among the rural and low educated groups [32]. This suggests the possibility that the validity and reliability of the GPAQ measure might also vary between different sub-populations. Third, the HCMC survey departed from the methods recommended in the STEPwise survey procedures by using reserve lists for replacing non-consenting or ineligible individuals. However, using the reserve lists was necessary to achieve the required sample size and reduced the possibility of survey staff conducting convenience sampling. Finally, we have followed the GPAQ analytical guidelines to calculate MET-minutes for physical activity. However, this made comparing our results with other studies difficult due to the different definitions of physical inactivity (weighting and scoring of physical activities) used. For example, although many studies used the common cut-off points of 30 minutes physical activity daily, this was applied to one physical activity domain, usually leisure time only.

Limitations aside, this study provides a valuable snapshot of physical activity patterns across three domains of physical activity for adults in HCMC, Vietnam, using standardised survey methodology and measures.

## Conclusion

With the rising burden of obesity and chronic diseases such as diabetes and cardiovascular disease, Vietnam will need to resource, develop and implement integrated preventive strategies to address physical inactivity induced by rapid motorisation and automation of work-related activities. At the individual level, an important consideration is identifying strategies for supporting the various population groups to continue to lead an active lifestyle. However, strategies aimed solely at increasing awareness and skills are unlikely to result in measurable behaviour change. Broader community-based and environmental-level policies for preserving active commuting especially, among older adults and promoting leisure-time physical activity across all ages and genders, especially to young adults, are also essential. To address this challenge, a comprehensive, multi-sectoral national plan of action on physical activity promotion for Vietnamese people is necessary as part of an integrated approach to preventing and controlling NCDs. This will also necessitate developing and communicating national-level recommendations on how much physical activity Vietnamese people would require for minimising cardiovascular and metabolic disease risks.

## Competing interests

The authors have no financial or personal relationships with other people or organizations that could inappropriately influence our work. The corresponding author has full access to all the data in the study and has final responsibility for the decision to submit for publication.

## Authors' contributions

NDN designed the study and supervised the project; TTHO conducted data collection, data analysis and prepared the manuscript; MJD, AEB and PP provided data analysis advice and preparation of the manuscript.

## Pre-publication history

The pre-publication history for this paper can be accessed here:


